# Dietary Intake of Selenium in Relation to Pubertal Development in Mexican Children

**DOI:** 10.3390/nu11071595

**Published:** 2019-07-14

**Authors:** Yun Liu, Karen E. Peterson, Brisa N. Sánchez, Andrew D. Jones, Alejandra Cantoral, Adriana Mercado-García, Maritsa Solano-González, Adrienne S. Ettinger, Martha M. Téllez-Rojo

**Affiliations:** 1 Department of Nutritional Sciences, University of Michigan School of Public Health, Ann Arbor, MI 48109, USA; 2 Department of Biostatistics, University of Michigan School of Public Health, Ann Arbor, MI, 48109, USA; 3 National Institute of Public Health, Cuernavaca 62100, Mexico

**Keywords:** selenium, dietary intake, Tanner stage, puberty, menarche

## Abstract

Alterations in pubertal timing have been associated with long-term health outcomes. While a few reports have shown that dietary intake of selenium is associated with fertility and testosterone levels in men, no human studies have considered the association between selenium and pubertal development in children. We examined the cross-sectional association of childhood dietary intake of selenium with pubertal development among 274 girls and 245 boys aged 10–18 years in Mexico City. Multiple logistic and ordinal regression models were used to capture the association between energy-adjusted selenium intake (below Recommended Dietary Allowance (RDA) vs. above RDA) and stages of sexual maturity in children, adjusted for covariates. We found that boys with consumption of selenium below the RDA had lower odds of a higher stage for pubic hair growth (odds ratio (OR) = 0.51, 95% confidence interval (95% CI): 0.27–0.97) and genital development (OR = 0.53, 95% CI: 0.28–0.99) as well as a lower probability of having matured testicular volume (OR = 0.37, 95% CI: 0.15–0.88) compared with boys who had adequate daily dietary intake of selenium (above RDA). No associations were found in girls. According to our results, it is possible that inadequate consumption of selenium may be associated with later pubertal development in boys, suggesting a sex-specific pattern. Future work with a larger sample size and measures of selenium biomarkers is needed to confirm our findings and improve understanding of the role of this mineral in children’s sexual development.

## 1. Introduction

Puberty, the transitional period between childhood and adult sexual maturity, is a complex process involving a series of biological events. Changes in the timing of pubertal onset have been observed over the past few decades, with studies from the US and Europe reporting a trend towards earlier menarche and thelarche in girls, as well as earlier genital development in boys [1]. Altered pubertal timing has received special attention due to its implications for the development of hormone-related cancers and chronic diseases in later life [2]. Nutritional factors during childhood have been shown to affect sexual maturation and account for 25% of the variation in pubertal timing [3]; inadequate childhood dietary intake of nutrients could negatively affect pubertal development [3,4].

Selenium is an essential trace element that plays an important role in animal and human health. The health effects of selenium are mostly mediated by selenoproteins (selenium-containing proteins) and antioxidant enzymes, such as glutathione peroxidase (GPx), thioredoxin reductase (TRxR) and iodothyronine deiodinase (DIO) [5]. Selenoproteins are shown to participate in the process of antioxidant defense, the regulation of thyroid hormones, the immune responses and reproduction [5]. Some human studies have suggested that selenoproteins may play a therapeutic role in some cancers [6]. High levels of selenium can be found in seafoods, organ meats and Brazil nuts. Other food sources include fish, dairy products, cereals and other grains [7]. Selenium exists in both inorganic and organic form, and both chemical forms can be consumed through diet [8]. Inorganic forms of selenium such as selenites and selenates can be found in soils and accumulated by plants and subsequently converted to organic forms (e.g., selenocysteine and selenomethionine). Selenomethionine is the major form of selenium that can be found in animal and human tissues [8,9]. The US Recommended Dietary Allowance (RDA) of selenium is 40 µg/d for children ≤13 years and 55 µg/d for children ≥14 years.

Animal studies have shown that selenium deficiency can cause reproductive problems [10]. In humans, low dietary selenium intake may cause infertility in men by affecting semen quality and sperm motility [11]. On the other hand, one of the largest intervention trials on infertile men revealed that selenium supplementation can increase testosterone levels [12]. To our knowledge, the only study that has examined the association between selenium and sexual development found that adequate intake of selenium or selenium supplementation during pregnancy in sheep affected several ovarian characteristics and reproductive functions in their female lambs but did not affect onset of puberty [13]. However, no study has considered these associations in humans.

Although the mechanisms of action involved in these relationships remain unclear, it is possible that inadequate intake of selenium could affect sexual development by interrupting the production of puberty-related hormones and levels of body fat, or by promoting oxidative stress, all of which have been linked to sexual maturation [14,15,16,17,18,19,20]. Since no epidemiological study has investigated the associations between selenium and pubertal development, the objective of this study was to examine whether childhood dietary intake of selenium is associated with pubertal development in a Mexican population of both boys and girls aged 10–18 years. We hypothesized that inadequate dietary intake of selenium is associated with later pubertal development in children.

## 2. Materials and Methods

### 2.1. Study Population

In this study, we included participants from the Early Life Exposures in Mexico to Environmental Toxicants (ELEMENT) project, which is an ongoing, 25 year longitudinal cohort of both pregnant women and their offspring in Mexico City. In 2015, child participants (*n* = 549) were invited to participate in a follow-up study visit based on the availability of archived maternal biological specimens. Pertinent details of the ELEMENT study including recruitment, eligibility criteria and collection of maternal information can be found elsewhere [21,22].

During the follow-up study, questionnaires regarding dietary intake of nutrients, anthropometric measures and data on Tanner stages were obtained from children. Among these 549 participants, 535 had data on at least one measurement of pubertal development. Of these 535 participants, 519 had complete information on the covariates of interest and were included in the final analysis.

Research protocols were approved by the Institutional Review Board at the University of Michigan and the Research, Biosafety and Ethics in Research committees at the National Institute of Public Health of Mexico (INSP). Prior to enrollment, informed consent was obtained from mothers and informed assent was obtained from their children.

### 2.2. Dietary Intake of Selenium

Daily dietary intakes of energy and selenium were estimated using a semi-quantitative food frequency questionnaire (FFQ) that was interviewer-administered by trained research staff. The questionnaire, adapted from the 2006 Mexican Health and Nutrition Survey [23], queried usual dietary intake of 109 foods in children over the previous week. Age-specific FFQs were applied separately to children aged 7–11 years and 12 years or older. Children aged 7–11 years (n = 88) were assisted by their mothers when needed to improve the accuracy and precision of dietary intakes. Values used to assess the frequency of usual intake ranged from “0” to “6 or more times per day,” which were then converted to a daily intake. The micronutrient composition of each item was obtained using a nutritional composition database of foods compiled by the National Institute of Public Health [24]. The daily intake of total energy (kcal) and selenium was calculated using a program developed by INSP staff by multiplying nutrient content in each food item with the frequency of reported usual intake of that item (servings per day) and then summing over the all food items containing that specific nutrient [25]. Given that only four children reported the use of selenium supplementation and the dosages were low, we did not include multivitamin use in this study. Intakes of selenium were adjusted for total energy intake using a nutrient residual model [26] to control for confounding effects and to remove extraneous variation. The intakes of selenium were then categorized into two groups according to the US Recommended Dietary Allowances (RDAs) classification (below and above RDA): the RDA of selenium is 40 μg/d for children ≤13 years and 55 μg/d for children ≥14 years.

### 2.3. Pubertal Measurement

A trained physician performed pubertal assessments using Tanner staging for breast and pubic hair growth in girls and for genitalia and pubic hair growth in boys on a scale of 1 (no sexual development) to 5 (full sexual development) [27,28]. Girls reported menarcheal status via a self-reported questionnaire, where girls aged 10–18 years were asked if and when they had started menstruation. Right and left testicular volumes were measured by the physician using a Prader orchidometer (range 1–25 mL). In this study, the larger volume of the right and left testicle was used. Testicular volume of 20 mL was applied to indicate matured stage (≥20 mL) [29].

### 2.4. Covariates

A body mass index (BMI) z-score for each child participant was calculated using the World Health Organization (WHO) standard growth curves specifically for children aged 5–19 years old, adjusting for children’s age and sex [30]. Child age and household socioeconomic status (SES) were obtained from questionnaires administered at the time of sexual assessment. The Mexican Association of Market and Public Opinion Research Agencies (AMAI) developed and standardized an index (i.e., AMAI 8 × 7) to classify the SES in the Mexican population. Seven socioeconomic levels ranging from A to E were identified based on the household assets: A/B and C+ indicate the upper class, while D and E represent the lower class. C, C− and D+ were defined as middle class [31]. Maternal age, marital status, smoking history and number of siblings at birth were collected from questionnaires at a pregnancy visit. It has been suggested that zinc with similar food sources of selenium may also be associated with pubertal development [32,33,34]; however, we did not include dietary intake of zinc in the models because zinc was not associated with puberty in our study sample.

### 2.5. Statistical Analysis

Descriptive statistics were calculated for each variable. The characteristics of participating children were compared by sex. T-tests were used to assess the differences between continuous variables, and Chi-square tests were adopted to examine the differences between categorical variables. A list of a priori variables, hypothesized to be potential confounders or known predictors of pubertal development were selected based on biological plausibility, including child age and BMI z-score, number of siblings at birth [35], maternal age, marital status [36] and smoking history, and household SES.

Multiple logistic and ordinal regression models were used to examine the associations of dietary intake of selenium (below RDA vs. above RDA) with indices of puberty in both sexes in separate models. Children’s usual daily intake of selenium estimated from the FFQ was treated as a primary predictor. For girls, ordinal Tanner staging for breast and pubic hair growth were considered as outcome variables. For boys, the amount of testicular volume (juvenile versus matured) and ordinal Tanner staging for genital and pubic hair growth were considered as outcome variables.

We used time-to-event (survival) analysis to study the association between daily dietary intake of selenium (below RDA vs. above RDA) and age at menarche in girls accounting for censored data [37]. The time to event (i.e., menarche) was based on the self-reported menarcheal age (years) or right-censored observations using girl’s age at the interview. Hazards ratios (HRs) and 95% confidence intervals (CIs) were obtained by using Cox proportional-hazard regression models. We defined statistical significance as *p* ≤ 0.05. All statistical analyses were performed using SAS statistical software (version 9.4; SAS Institute Inc., Cary, NC, USA).

## 3. Results

Among 519 participants (274 girls and 245 boys), the mean age was 13.9 years, ranging from 10 to 18 years (Table 1). On average, children’s BMI z-score was 0.5, and they had two siblings. Their mothers were 26.4 (SD = 5.4) years old at delivery, 28.8% were single, and 47.7% had a history of maternal smoking. None of the demographic characteristics of participants differed significantly by children’s sex. There were 217 girls and 197 boys who had dietary intake of selenium below the RDA. As shown in Table 2, among 245 boys with available data on puberty, 19.2% were at Stage 1 (pre-pubertal) and 20.4% were at Stage 5 (adult level) for pubic hair growth; 5.3% and 21.6% were at Stage 1 and 5 for genitalia, respectively. There were 171 boys (69.8%) who had matured testicular volume (≥20 mL). Among 266 girls with available data on Tanner staging, 6.8% were at Stage 1 and 19.6% were at Stage 5 for pubic hair growth; 4.1% and 23.3% were at Stage 1 and 5 for breast maturation, respectively. Among 271 girls (84.9%) who had attained menarche, the mean (SD) age at menarche was 11.5 (SD = 1.2) years.

Our data showed no significant associations between energy-adjusted childhood consumption of selenium and sexual development in girls (Table 3). We found that low dietary intake of selenium with energy adjustment was associated with later pubertal development associations in boys, adjusted for child age and BMI z-score, number of siblings at birth, maternal age, marital status and smoking history, and household SES (Table 4). Specifically, boys with inadequate daily dietary intake of selenium (below RDA) had lower odds of a higher stage for pubic hair growth (odds ratio (OR) = 0.51, 95% confidence interval (95% CI): 0.27–0.97, *p* = 0.039) and genital development (OR = 0.53, 95% CI: 0.28–0.99, *p* = 0.049) compared with boys who had adequate daily dietary intake of selenium (above RDA). Similarly, boys with low consumption of selenium had a lower probability of having a matured testicular volume (OR = 0.37, 95% CI: 0.15–0.88, *p* = 0.024) compared with boys who had adequate intake of selenium.

## 4. Discussion

We examined the cross-sectional associations between dietary consumption of selenium and sexual maturity in children at age 10–18 years in Mexico City. Inadequate intake of selenium was associated with later pubertal development in boys, while no significant associations were found in girls, suggesting a differential effect of selenium on pubertal development by sex. Although the mechanism by which dietary selenium consumption during childhood may affect puberty is largely uncharacterized, selenium may influence pubertal development through several pathways including its involvement in the production of sex hormones, growth hormones, body fat and oxidative stress as these factors have been shown to play an important role in the process of sexual maturation [14,15,16,17,18,19,20].

It is well known that the growth of pubic hair is highly androgen-dependent, primarily regulated by dehydroepiandrosterone (DHEA)/dehydroepiandrosterone sulfate (DHEA-S) [38]. It has been reported that DHEA may reduce body fat mass and increase muscle mass in normal men [39]. Other studies found that the effects of DHEA-S on body fat distribution were sex-related by showing a negative association of DHEA-S levels with visceral fat in men and with abdominal body fat in women [40]. There is also evidence that selenium may affect body composition by decreasing body mass index (BMI), waist circumference (WC) and percentage of body fat (% BF) in adults [41,42]. Taken together, it is reasonable to hypothesize that inadequate selenium levels may be associated with increased body composition and fat distribution in children, which then decrease DHEA/DHEAS levels, eventually leading to delayed pubic hair growth. Finally, it has been recognized that the body composition of pre-pubertal children varies by sex. Prior to pubertal onset, compared to boys at the same age, weight and height, girls have lower fat free mass (FFM) and higher % BF [43]. This difference may explain the varying effect of selenium intake on pubic hair growth by sex observed in this study.

Insulin-like growth factor 1 (IGF-1) has been shown to promote pubertal development with increased serum testosterone levels, pituitary luteinizing hormone content [44] and testicular growth in male animals [45]. A clinical trial study of 443 elderly healthy Swedish participants found that the levels of IGF-1 increased in participants who received active selenium supplementation compared with those who received a placebo [46]. Furthermore, the concentration of IGF-1 decreased for participants with a relatively low selenium status at baseline who received no selenium supplementation [46]. Although the precise underlying mechanism remains poorly understood, it is possible that the observed decreased IGF-1 levels may be related to the increase in both inflammatory responses and oxidative stress possibly resulting from low selenium levels [47], which subsequently delay the development of genitalia and decrease the levels of testicular volume.

In the present study, we observed significant associations of low selenium intake with later pubertal development in boys. However, we did not observe a significant association between selenium and sexual development in girls, which is consistent with the results from an animal study [13]. In that study, 43 ewes were randomly assigned to receive selenium treatment during pregnancy; they received either adequate selenium (9.5 mg/kg body weight) or high selenium (81.8 mg/kg body weight). Ovarian and uterine weight and pubertal onset were assessed in their female adolescent offspring. The authors found that maternal selenium intake (diet with adequate vs. high selenium) did not affect uterine or ovarian weight and onset of puberty in female offspring [13]. No previous studies have examined the association between selenium and pubertal development in males. Selenium is an essential component of selenoproteins; selenoproteins play an important role in antioxidant defense, highly expressed in spermatids and exhibit high activity in postpubertal testis in rodents [48,49]. Earlier animal studies have shown that selenium promotes male fertility and the deposition of selenium is higher in testes compared with other organs [50]. During puberty, the selenium levels in testes largely increase [51]. Under selenium compromised conditions, testes compete with the brain to obtain more selenium [52]. The majority of our study participants consumed inadequate selenium (below the RDA). This possibly because the levels of dietary intake of selenium are associated with the selenium content in soils. It has been shown that the soils in Mexico have low amounts of selenium, which leads to low selenium levels in plants and animals [53].

One potential limitation of our analyses is that we did not have biomarkers of selenium (e.g., plasma) to relate to pubertal outcomes in children in this population. Our study is also limited by the use of a single FFQ which may not provide the most accurate information on dietary intake compared with other assessment tools (e.g., multiple 24 h recalls and 7 day food records). The decision to use FFQ was based on the budget and the intensity of participants’ involvement. However, our study has several strengths compared to previous reports. This is the first epidemiological study that examines the effects of dietary intake of selenium during childhood on physician-assessed pubertal development in children. Additionally, our analyses provide insights into how selenium intake may influence sexual maturity in both sexes. We found some evidence suggesting that selenium may be associated with various physical markers of pubertal development such as the growth of pubic hair, genitalia and testicular volume in boys living in Mexico City.

## 5. Conclusions

In our study, dietary intake of selenium below the RDA was associated with later development of pubic hair, genitalia and testicular volume in Mexican boys, while no associations were observed in Mexican girls. Our research is the only human study that directly examines the association between selenium and puberty with a few limitations. Results from future prospective studies with larger sample size and biomarkers of selenium status are warranted to confirm these findings and to promote the understanding of the role of nutritional factors in puberty.

## Figures and Tables

**Table 1:** Demographic characteristics and selenium consumption of participants living in Mexico City.

Characteristics	Overall (*N* = 519)	Boys (*N* = 245)	Girls (*N* = 274)
**Children**			
Age (year)	13.9 (2.1)	13.9 (2.0)	13.9 (2.1)
BMI z-score	0.5 (1.2)	0.4 (1.3)	0.6 (1.2)
Number of siblings at birth	2.0 (1.0)	2.0 (1.0)	2.0 (1.0)
**Mothers**			
Maternal age (year)	26.4 (5.4)	26.1 (5.4)	26.6 (5.5)
Marital status			
Yes	376 (71.2%)	178 (72.7%)	192 (70.1%)
No	152 (28.8%)	67 (27.3%)	82 (29.9%)
Smoking history			
Ever	248 (47.7%)	117 (47.8%)	131 (47.8%)
Never	272 (52.3%)	128 (52.2%)	143 (52.2%)
Household socioeconomic status			
Lower class	144 (27.2%)	59 (24.3%)	81 (29.9%)
Middle class	354 (66.8%)	168 (69.1%)	176 (64.9%)
Upper class	32 (6.0%)	16 (6.6%)	14 (5.2%)
**Selenium**			
dietary intake (μg/d) *	35.8 (25.1, 32.6, 42.5, 52.8)	35.9 (24.4, 31.7, 41.7, 55.1)	35.7 (25.3, 34.0, 42.8, 51.7)

BMI, body mass index. * denotes the values for mean, p25, p50, p75 and p90.

**Table 2:** Distribution of physician-assessed secondary sex characteristics ^a^.

Measure	Stage	*N* (%)
Boys		
Pubic hair	1	47 (19.2)
	2	31 (12.7)
	3	63 (25.7)
	4	54 (22.0)
	5	50 (20.4)
Genitalia	1	13 (5.3)
	2	33 (13.5)
	3	43 (17.6)
	4	103 (42.0)
	5	53 (21.6)
Testicular volume	Yes (≥20 mL)	171 (69.8)
	No	74 (30.2)
Girls		
Pubic hair	1	18 (6.8)
	2	65 (24.4)
	3	63 (23.7)
	4	68 (25.6)
	5	52 (19.6)
Breast	1	11 (4.1)
	2	26 (9.8)
	3	69 (25.9)
	4	98 (36.8)
	5	62 (23.3)
Menarche	Yes	230 (84.9)
	No	41 (15.1)

^a^ The sample size is 245 for each indicator of puberty in boys. For girls, 266 girls had available data on pubic hair and breast development, and 271 girls had data on menarche.

**Table 3:** Adjusted associations between dietary intake of selenium (below RDA vs. above RDA) and physician-assessed secondary sex characteristics in girls at age 10–18 years.

Variable	Pubic Hair ^a^		Breast ^a^		Menarche ^b^	
	OR (95% CI)	*p*-Value	OR (95% CI)	*p*-Value	HR (95% CI)	*p*-Value
Dietary intake of selenium (below RDA vs. above RDA)	0.98 (0.52, 1.86)	0.951	1.34 (0.70, 2.57)	0.377	0.90 (0.62, 1.33)	0.606
Child Age	2.40 (2.06, 2.80)	<0.0001	2.52 (2.13, 2.97)	<0.0001	-	
Child BMI z-score	1.35 (1.10, 1.64)	0.004	1.49 (1.21, 1.84)	0.000	1.22 (1.08, 1.36)	0.001
Number of siblings at birth	0.94 (0.73, 1.22)	0.658	0.99 (0.77, 1.29)	0.975	0.86 (0.74, 1.00)	0.046
Maternal age	1.03 (0.98, 1.08)	0.179	0.99 (0.95, 1.05)	0.883	1.00 (0.97, 1.03)	0.917
Maternal marital status (reference: single)	0.46 (0.27, 0.79)	0.004	0.49 (0.28, 0.84)	0.010	0.76 (0.56, 1.03)	0.073
Maternal smoking history (reference: never)	1.24 (0.77, 1.99)	0.371	1.06 (0.65, 1.72)	0.825	1.15 (0.87, 1.52)	0.340
Household socioeconomic status (reference: lower class)						
Middle class	1.19 (0.71, 2.02)	0.694	1.58 (0.92, 2.72)	0.857	0.78 (0.57, 1.07)	0.129
Upper class	1.84 (0.58, 5.76)	0.347	2.83 (0.86, 9.35)	0.160	0.96 (0.51, 1.82)	0.910

CI, confidence interval; HR, hazard ratio; OR, odds ratio; RDA, Recommended Dietary Allowance. ^a^ For pubic hair and breast, all estimates are from ordinal regression models. For testicular volume, all estimates are from logistic regression models. All models adjusted for child age and BMI z-score, number of siblings at birth, maternal age, marital status and smoking history, and household socioeconomic status. ^b^ All estimates are from Cox proportional-hazard models adjusted for child BMI z-score, number of siblings at birth, maternal age, marital status and smoking, and household socioeconomic status.

**Table 4:** Adjusted associations between dietary intake of selenium (below RDA vs. above RDA) and physician-assessed secondary sex characteristics in boys at age 10–18 years ^a^.

Variable	Pubic Hair		Genitalia		Testicular Volume	
	OR (95% CI)	*p*-Value	OR (95% CI)	*p*-Value	OR (95% CI)	*p*-Value
Dietary intake of selenium (below RDA vs. above RDA)	0.51 (0.27, 0.97)	0.039	0.53 (0.28, 0.99)	0.049	0.37 (0.15, 0.88)	0.024
Child Age	3.33 (2.74, 4.06)	<0.0001	2.48 (2.08, 2.95)	<0.0001	2.33 (1.84, 2.94)	<0.0001
Child BMI z-score	1.01 (0.83, 1.23)	0.887	0.81 (0.67, 0.99)	0.037	1.34 (1.03, 1.74)	0.031
Number of siblings at birth	0.72 (0.56, 0.92)	0.010	0.81 (0.63, 1.03)	0.087	0.94 (0.67, 1.32)	0.710
Maternal age	1.03 (0.98, 1.09)	0.206	1.05 (0.99, 1.10)	0.083	0.97 (0.90, 1.03)	0.302
Maternal marital status (reference: single)	0.69 (0.39, 1.20)	0.187	0.93 (0.53, 1.61)	0.788	0.88 (0.41, 1.88)	0.739
Maternal smoking history (reference: never)	0.84 (0.51, 1.39)	0.505	0.80 (0.49, 1.32)	0.393	0.93 (0.47, 1.84)	0.832
Household socioeconomic status (reference: lower class)						
Middle class	0.97 (0.54, 1.72)	0.506	1.39 (0.78, 2.47)	0.052	0.93 (0.41, 2.08)	0.679
Upper class	0.61 (0.21, 1.81)	0.358	0.56 (0.19, 1.65)	0.146	0.59 (0.12, 2.83)	0.512

CI, confidence interval; RDA, Recommended Dietary Allowance. ^a^ For pubic hair, genitalia and breast, all estimates are from ordinal regression models. For testicular volume, all estimates are from logistic regression models. All models adjusted for child age and BMI z-score, number of siblings at birth, maternal age, marital status and smoking history, and household socioeconomic status.
